# Non-cognate ligands of hepatitis C virus envelope broadly neutralizing antibodies induce virus-neutralizing sera in mice

**DOI:** 10.3389/fimmu.2025.1624299

**Published:** 2025-07-22

**Authors:** Stephen Ian Walimbwa, Shiv Bharadwaj, Petr Kosztyu, Lucie Vankova, Milan Kuchar, Eliska Kopecna, Roman Effenberg, Lukas Drasar, Leona Raskova Kafkova, Petr Maly, Milan Raska

**Affiliations:** ^1^ Department of Immunology, Faculty of Medicine and Dentistry, Palacky University Olomouc and University Hospital, Olomouc, Czechia; ^2^ Infectious Diseases Institute, Makerere University College of Health Sciences, Kampala, Uganda; ^3^ Laboratory of Ligand Engineering, Institute of Biotechnology of the Czech Academy of Sciences, BIOCEV Research Center, Vestec, Czechia; ^4^ Department of Chemistry of Natural Compounds, University of Chemistry and Technology, Prague, Czechia

**Keywords:** hepatitis C, vaccine, mimotope, broadly neutralizing antibodies, myomedins, protein mimicry, protein scaffolds

## Abstract

**Introduction:**

The persistent rise in new Hepatitis C virus (HCV) infections threatens WHO efforts to eliminate HCV infection by 2030. Although direct-acting antiviral (DAA) drugs are efficacious, access remains limited, reinfections occur, and perinatal infections continue to pose long-term complications. Therefore, an effective anti-HCV vaccine is urgently needed.

**Methods:**

We employed a highly complex combinatorial Myomedin-loop scaffold library to identify variants binding to paratopes of HCV E2-specific broadly neutralizing antibodies (bNAbs) HC-1AM and HC84.26.WH.5DL. The selected binders, named SHB and WIN, respectively, represent non-cognate mimotopes of the aforementioned bNAbs. These binders were subsequently used as immunogens in experimental mice to elicit serum antibodies capable of binding to HCV E2 and neutralize HCV pseudotyped viruses.

**Results and discussion:**

The non-cognate mimotopes SHB and WIN competed with the E2 glycoprotein for bNAbs binding and, after immunizing experimental mice, elicited E2- and HCV-pseudovirus-specific antibodies. WIN- and SHB-immunized mice exhibited neutralization against 15 HCV pseudoviruses with varying neutralization sensitivities. The most potent binders WIN028 and WIN047, were modified with a C-terminal His-tag, allowing the generation of WIN proteoliposome and subsequent use in experimental mice immunizations. Hyperimmune sera exhibited improved binding to HCV E2 and neutralized 60% of the tested HCV pseudoviruses. The broad neutralization of HCV pseudoviruses achieved by hypperimmune sera from SHB- and WIN-immunized mice highlights the potential of this approach in the HCV vaccine design.

## Introduction

Hepatitis C virus (HCV) infection is a significant public health concern, affecting approximately 0.7% of the global population (1, 2). Acute infections are typically asymptomatic and can progress to chronic HCV, potentially leading to liver cirrhosis or hepatocellular carcinoma, which are the major indications for liver transplantation (3).

Direct-acting antivirals (DAAs) cure most infections and are the standard of care for treating chronic HCV with a robust ability to induce sustained virologic responses (4–6). However, the development of advanced liver disease is often preceded by nonspecific clinical manifestations that are seldom attributed to chronic HCV (7). People living with chronic HCV are usually diagnosed late, presenting with signs of end-stage liver injury and significant hepatic remodeling, which directly impacts the efficacy of DAAs (5, 7).

Restricted access to DAAs due to multiple public health and financial constraints has resulted in persistent global infections (8). Increases in annual infections continue to be reported in the most at-risk populations and in patients living in resource-constrained countries. Consequently, the WHO target of eliminating viral hepatitis as a public health threat by 2030 will not be achieved if barriers to early detection and access to DAAs are not addressed (2, 8). Furthermore, without the development of an effective HCV vaccine, preventive measures to curb the spread of the HCV will remain challenging.

To date, efforts to develop an efficacious vaccine against HCV have been unsuccessful. HCV is an enveloped, positive-sense, single-stranded RNA virus (+ssRNA virus) belonging to the genus *Hepacivirus* in the family *Flaviviridae* (9–11). The HCV genome is 9.6 kb and contains a single open reading frame encoding structural glycoproteins (Core, E1, and E2) and non-structural glycoproteins (NS2, NS3, NS4A, NS4B, NS5A, and NS5B) that are translated via an internal ribosomal entry site. The glycoproteins are cleaved in the endoplasmic reticulum by cellular peptidases and viral proteases (9, 12). HCV replication and assembly are mediated by a non-proofreading RNA-dependent RNA polymerase (RdRp) that generates quasispecies in infected individuals. This, together with the host immune responses, facilitates the development of escape mutants (5, 9). Notably, RdRp coupled with the rapid viral replication creates the enormous genetic diversity exhibited by HCV. Currently, eight genotypes and over 100 subtypes, whose nucleotide sequences differ by approximately 30% and 15% respectively, have been reported (3, 8). This high sequence diversity of the E1 and E2 structural glycoproteins poses a major barrier to the development of an effective HCV vaccine (1, 13).

HCV viral entry into liver hepatocytes is a highly coordinated multi-step process that remains poorly understood. The E1-E2 structural glycoproteins mediate fusion between the viral membrane and the hepatocyte endosomal membrane through interactions with viral attachment receptors, including low density lipoproteins, apolipoproteins, and heparan sulfate proteoglycans (14). Although evidence suggests that E1 is responsible for membrane fusion, viral internalization is preceded by conformational changes in E2 glycoprotein driven by interactions with the scavenger receptor BI and tetraspanin CD81. The formation of the HCV–CD81 complex initiates signaling and membrane diffusion towards hepatocyte tight junctions through claudin-1 and occludin-mediated endocytosis (14, 15).

E2 is the primary target for HCV broadly neutralizing antibodies (bNAbs) and is relatively conserved across HCV strains (15–17). Crystal structures of E2 have characterized several distinct antigenic sites, referred to as antigenic regions (AR1-5) or domains I-III, through which the immune system neutralizes the virus. bNAbs predominately target the antigenic region 3 (AR3) to neutralize HCV by preventing E2–CD81 interactions and blocking adjacent epitopes (14, 15). Rational vaccine strategies to develop HCV vaccines have focused on replicating the mechanisms deduced from AR3–bNAbs interactions (16). Although extensive vaccine research has been focused on inducing antibodies targeting the E2 linear epitopes in the AR3, vaccine efficacy has been hampered by non-neutralizing antibodies targeting non-conserved epitopes in AR1 and AR2 (18). Here, we report on a reverse vaccinology approach for developing HCV vaccines based on the protein mimicry concept. We recently demonstrated that small binding proteins derived from scaffolds of the Albumin Binding Domain and human Myomesin-derived Myomedin-loop binders specifically recognized paratopes of HIV-1 bNAbs VRC-01, 10E8, PGT121, and PGT126. These proteins mimic natural epitopes located in specific HIV-1 Env regions and, when used as immunogens, stimulate the production of Env-specific antibodies. Murine hyperimmune sera elicited by these immunogens neutralized HIV-1 clades A, B, and C pseudoviruses *in vitro*. This approach, termed the “Non-cognate ligand strategy” (NCLS) (19–21), was employed here to select protein imprints of the E2 AR3 supersite domains B and D, with enhanced potency and neutralization breadth targeting paratopes of HCV bNAbs HC-1AM and HC84.26.WH.5DL. These bNAbs prevent conformational changes to the E1-E2 heterodimer and block viral entry by inhibiting binding to the CD81 co-receptor. Notably, in cell cultures systems at appropriate IC_50_ concentrations, no viral escape mutants have been observed for domain B- and D-specific neutralizing human monoclonal antibodies. Therefore, these bNAbs represent promising targets for vaccine development (22, 23). The small binding proteins utilized in this study elicited antibodies recognizing the HCV E2 surface glycoprotein in experimental BALB/c mice. *In vitro*, the vaccine-induced anti-E2 immunoglobulin G (IgG) antibodies effectively neutralized 50% of the tested HCV pseudovirus panel. Thus, these binders provide insight into developing novel prophylactic vaccine candidates using non-cognate ligands.

## Methods

### Myomedin combinatorial loop-library assembly

The Myomedin-loop library was designed and compiled using a series of polymerase chain reactions (PCR) with Phusion High-Fidelity DNA Polymerase (NEB, Massachusetts, USA), as previously reported (19, 21).

### Anti-HCV antibodies

Neutralizing anti-HCV monoclonal antibodies HC-1AM and HC84.26.WH.5DL (HC84), as well as the anti-human cytomegalovirus (HCMV) antibody R04, were kindly provided by Prof. Steven Foung, Standford University, USA. HC-1AM IgG1λ and HC84 IgG1κ were used as target proteins during directed evolution by ribosome display (RD) and in enzyme-linked immunosorbent assay (ELISA) applications. Human IgG1 with λ light chain (Sigma-Aldrich, St. Louis, MO) or R04 were used as an isotype control in the library preselection during ribosome display (RD) and as isotype controls in ELISA. All experiments were performed using secondary antibodies and antibody-conjugates of molecular grade.

### Ribosome display

The combinatorial Myomedin-loop library was processed through four rounds of *in vitro* transcription/translation and ribosome display selection, as previously reported (21). Briefly, 96-well MaxiSorp plates (Thermo Scientific™ Clear Flat-Bottom Immuno Nonsterile 96-Well Plates) were coated with a constant concentration (25 µg/ml) of human IgG1λ type or R04 during the preselection of the library. Using varied concentrations (first round: 25 µg/ml, second round: 10 µg/ml, third round: 10 µg/ml, and fourth round: 5 µg/ml) of either anti-HCV HC-1AM or HC84 neutralizing antibodies in 100 mM bicarbonate/carbonate buffer (pH 9.6), we adjusted the stringency during each round of RD selection. Also, to further impose stringency during each round, different concentrations of Tween 20 in phosphate-buffered (PBST, pH 7.2), i.e., 0.05% Tween/5 times, 0.05% Tween/10 times, 0.05% Tween/10 times, and 0.25% Tween/10 times were used in the first, second, third and fourth cycle of RD selection, respectively. The complementary DNA (cDNA) from the fourth round of RD selection was amplified into double-stranded DNA via PCR using primers JOIN-F(CTATAGGGAGACCACAACGGTTTCCCTCTAGAAATAATTTTGTTTAACTTTAAGAAGGAGATATACATATGAAAAGCGAGCTGGCCG) and JOIN-R (GAACCGACCGCGGATCCACCCTGTTTACGAATCCATTCTT). The PCR product was further amplified with primers His-Myo-F (CAGTCCATGGGCAGCAGCCATCATCATCATCATCACAGCAGCGGCAAAAGCGAGCTGGCCG) and JOIN-R to insert cloning sites, as previously reported (21). Finally, the amplified combinatorial Myomedin-loop library DNA was digested using NcoI and BamHI restriction enzymes and fused with the V5-tag sequence into a pET-28b cloning vector. The resulting plasmid library was then used to transform *Escherichia coli* (*E. coli)* XL1-Blue cells. Individual colonies expressing full-length Myomedin-loop variants were selected for further analysis.

### Myomedin-loop variants production

Myomedin-loop variants (~16 kDa) containing a hexahistidine (His_6_)-tag at the N-terminus and a V5-tag at the C-terminus (His_6_-Myomedin-V5) were produced in *E. coli* BL21 (DE3) cultured in Luria-Bertani (LB) broth supplemented with kanamycin (60 µg/ml). Briefly, the culture was incubated at 37°C with continuous shaking (230 rpm) until an optical density at 600 nm (OD_600_) of ~0.6 was reached. Protein expression was induced with 1 mM IPTG, followed by incubation for an additional 4h at 32°C. Bacterial cells were harvested by centrifugation (6,000 × g, 4°C, 15 min) and the pellet was resuspended in TN buffer (50 mM Tris, 150 mM NaCl, pH 8.0). The suspension was disrupted using a sonicator (for 10 min with 10 sec on/10 sec off cycle; Misonix Sonicator^®^ 3,000 Ultrasonic Cell Disruptor), and the supernatant containing soluble protein was collected by centrifugation (40,000 × g, 4°C, 20 min),. Bacterial lysates were either used directly or mixed with Ni-NTA agarose matrix for protein isolation prior to ELISA.

### ELISA and competition ELISA

All ELISA assays were performed using 96-well MaxiSorp plates (Thermo Scientific™ Clear Flat-Bottom Immuno Nonsterile 96-Well Plates). Bacterial lysates or purified protein were used to screen for Myomedin variants binding to anti-HCV antibodies in comparison to an isotype control (human IgG1λ type or anti-HMCV R04 antibody). In this assay, the MaxiSorp plate was coated overnight with 1 or 2 μg/ml of anti-HCV antibody in 100 mM bicarbonate/carbonate buffer (pH 9.6). The following day, the plate was washed with PBST (0.05% Tween-20, pH 7.4), and blocked with protein-free blocking buffer (PFBB) for 2 h at room temperature (RT). Then 50-fold diluted bacterial lysates or purified proteins (2 μg/ml) in PBST with 1% BSA (PBSTB) were applied and incubated for 1 h at RT. Specific Myomedin-loop variants were detected using anti-V5-tag–horseradish peroxidase (HRP) conjugated antibody (Abcam, Cambridge, UK) in PBSTB (1:10,000 dilution). For the competition assay, E2 protein (0.25, 0.5, or 1 µM) was serially diluted in PBSTB as a competitor of Myomedin variants (His-Myo-V5) used at constant concentration (0.5 or 0.3 µM). After incubation for 1h at RT, Myomedin variants were detected by anti-V5-tag–HRP conjugated antibody (Abcam, Cambridge, UK) in PBSTB (1:10,000 dilution). Cross-reactivity of the selected Myomedin-loop variants was also determined using concentration-dependent binding with non-specific anti-HCV antibodies under similar conditions. For each ELISA setup, a colorimetric signal was observed after 15 min of incubation as a reaction of horseradish peroxidase (HRP) with TMB (3, 3’, 5, 5’-tetramethylbenzidine)-Complete 2 substrates (TestLine Clinical Diagnostics s.r.o., Brno, Czech Republic) at RT. This reaction was stopped with 2 M sulfuric acid, and the absorbance at 450 nm was measured using a Multi-Mode ELISA Reader (BioTek Synergy™ HTX system).

### Experimental mice and Immunization

#### Ethical statement

All the animal experiments were conducted in accordance with the regulations and guidelines of the Czech Animal Protection Act (No. 246/1992) and were approved by the Animal Welfare Committee of the Faculty of Medicine, Dentistry of Palacky University Olomouc and the Ministry of Education, Youth and Sports, Czech Republic under protocol MSMT-22633/2022-6.

Healthy female 6- to 8-week-old BALB/c mice (AnLab, Brno, Czech Republic) were used in all experiments. Mice were housed under standard conditions following ARRIVE guidelines. All mice experiments were approved by the Ethics Committee of the Faculty of Medicine and Dentistry (Palacky University Olomouc, Czech Republic), and the Ministry of Education, Youth and Sports, Czech Republic (MSMT-22633/2022-6).

For soluble binder immunizations, 10 µg of the binder diluted in 50 µl of sterile PBS (total 100 µl volume per mouse) was mixed 1:1 with Freund´s adjuvant (Sigma Aldrich, St. Louis, MO, USA). For proteoliposome immunizations, N- or C-His-tagged WIN binders (10 µg per dose) were mixed with 100 µg (per dose) of preformed EPC/POPG/DOGS-NTA-Ni liposomes (EPC: POPG: DOGS-NTA-Ni of 76:19:5 mol%) as modified formulations from a previously described experiment (24) and adjuvanted with Monophosphoryl lipid A (MPLA; 32.6 µg per dose). Each mouse was administered 100 µl of the immunogen via an intradermal route, following a prime and 3X-boost regimen.

Blood samples from the lateral tail vein incision were collected from conscious mice before the prime, second, and third boost injections. For the terminal bleed, two weeks after the last boost, mice were anesthetized with ketamine/xylazine (100 mg/kg + 5 mg/kg in 100 µl of PBS, intraperitoneally). Blood was collected from the axillary plexus, and mice were immediately euthanized by cervical dislocation (25).

### ELISA for HCV E2-specific serum IgG antibodies

The reactivity of immunized mice sera to HCV E2 protein (R&D Systems, Inc., Minneapolis, MN, USA) was determined by ELISA. 96-well plates were precoated overnight with E2 protein (100 ng/well) at 4°C. After washing and blocking with 1% BSA/PBS/Tween-20 for 3 h at RT, sera were diluted in blocking buffer, added in duplicates, and incubated overnight at 4°C. After washing, bound antibodies targeting the E2 protein were detected by incubating with rabbit anti-mouse IgG secondary antibody HRP conjugate (Sigma-Aldrich, St. Louis, MO) diluted in blocking buffer for 3 h at RT. The binding signal was developed by O-phenylenediamineH_2_O_2_ substrate, and the reaction was stopped by adding 1 M sulfuric acid. Absorbance was measured at 492 nm.

### ELISA for HCV pseudotyped virus-specific serum IgG antibodies

Pseudotyped viruses were ultracentrifuged, and the pellet was resuspended in PBS. 96-well plates were coated with resuspended pseudotyped viruses overnight at 4°C. After washing with PBS, the plates were blocked in 3% BSA/PBS. Sera were serially diluted in the blocking buffer and incubated overnight at 4°C. After washing with PBS, an anti-mouse IgG secondary antibody HRP conjugate (Sigma-Aldrich, St. Louis, MO) diluted in blocking buffer was added and incubated for 1 h at RT. A signal was developed using O-phenylenediamin-H_2_O_2_ substrate and stopped with 1M sulfuric acid. Absorbance was measured at 492 nm.

### HCV pseudotyped virus production

To produce pseudotyped viruses, HEK293/17 cells at 60–90% confluency in 25 cm^2^ culture flasks were co-transfected using ViaFect transfection reagent (Promega, Madison, WI, USA). Before transfection, 10 μg of pNL4.3.Luc.R-E plasmid (NIH AIDS Research and Reference Reagent Program, Division of AIDS, NIAID, NIH), 1 µg of E1E2 expression plasmid (a gift from Jonathan Ball, Addgene, Watertown, MA, USA), and 22 µl of ViaFect were mixed with Dulbecco’s modified Eagle’s medium (DMEM) to a total volume of 300 µl and incubated for 30 min at RT. Then, the transfection mixture was added to 5 ml of DMEM in a flask with HEK293/17 cells. After 5 h incubation, fresh culture medium was added to the flasks, and cells were incubated for an additional 48 h. HCV pseudoviruses were harvested, aliquoted, and stored at -80°C.

### Virus neutralization assay

Genotypes 1–5 pseudoviruses were used for the neutralization assay in the human hepatoma cell line Huh7. Cells were seeded into 96-well plates and incubated overnight. Then, 50 μl of pseudoviruses were mixed with 5 µl of diluted serum (starting dilution 1:30) in 70 µl of DMEM and titrated by factor 3. After 90 min of incubation at 37°C, the serum and virus mixture were added to the Huh7 cells. The next day, fresh medium was added, and the plates were further incubated at 37°C in 5% CO_2_ for 72 h. After removing the culture medium, cells were lysed with 50 µl of Lysis buffer containing luciferin (Promega) for 2 min. 40 µl of the lysate were transferred into black 96-well plates, and luminescence was measured using an HP luminometer.

### Statistics

Statistical differences between groups were calculated using the Kruskal-Wallis analysis of variance (ANOVA) with Dunn´s *post hoc* test. Statistical significance was set at *p* < 0.05. All statistical analyses were performed using SPSSv.21 statistical packages (IBM Corp., Armonk, NY, USA) or GraphPad Prism 5 Software (GraphPad Software Inc., San Diego, CA, USA).

## Results

### Ribosomal display and selection of anti-E2 Myomedin mimotopes

**Figure 1:**
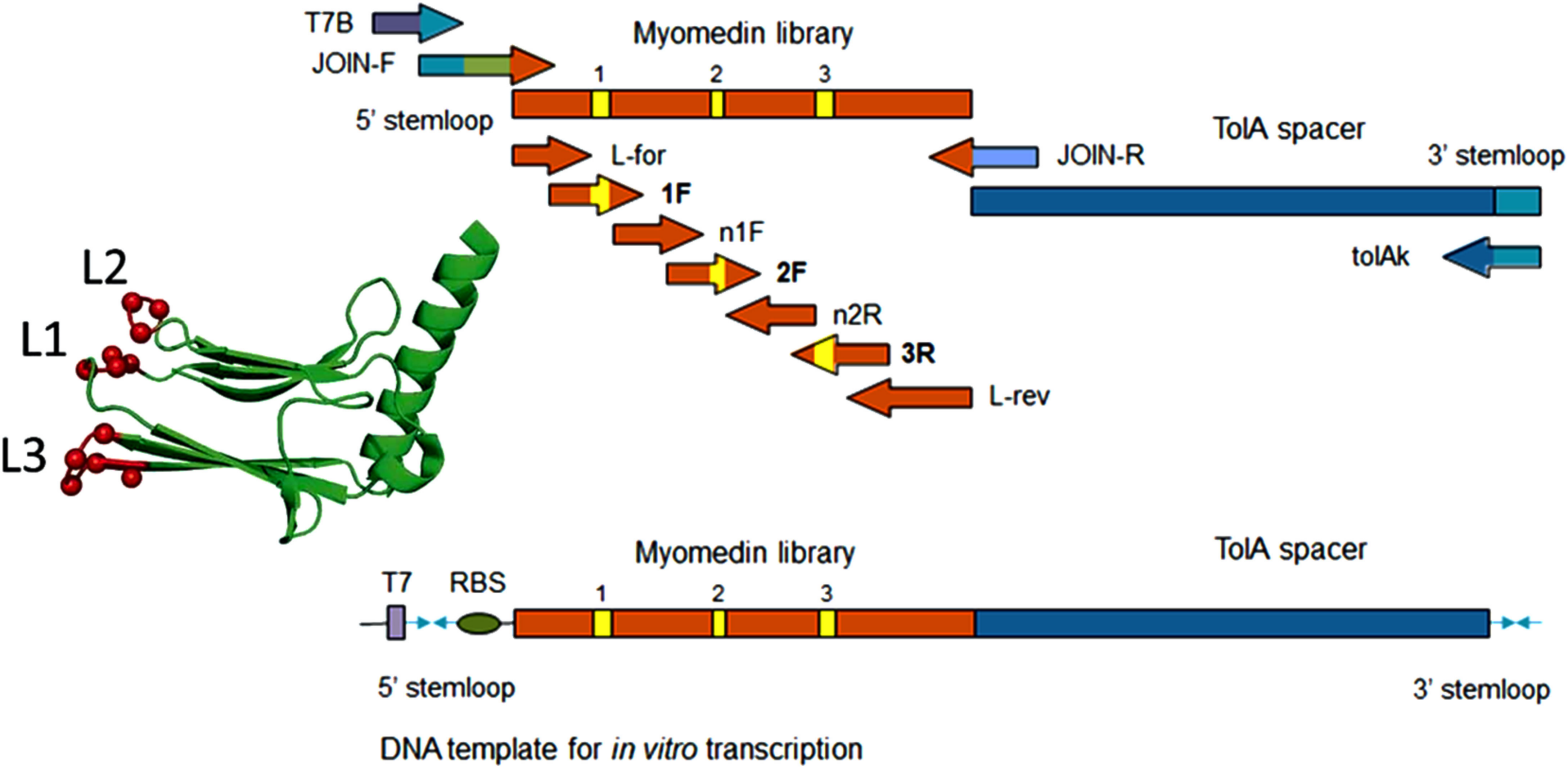
Scheme of Myomedin loop variant library assembly using multistep PCR showing the forward and reverse primers.

We assembled a combinatorial Myomedin library carrying 12 mutable amino acid residues located in the loop region using multiple PCR reactions (**Figure 1**). The randomization of codons was achieved via selected primers incorporating preassembled trinucleotide building blocks, employing trinucleotide mutagenesis (TRIM) technology. This approach facilitated complete customization of the amino acid composition at randomized sites, preventing the occurrence of unwanted stop codons or amino acids, including exclusion of cysteine residues to prevent the formation of disulfide bridges. To identify the Myomedin-loop scaffold proteins that mimic specific E2 epitopes recognized and targeted by HCV bNAbs HC-1AM (Domain B) and HC84 (Domain D), four rounds of ribosome display (RD) were performed on a Maxisorp plate. After the 4^th^ round, DNA transcripts were cloned into a pET28b cloning vector, transformed into *E. coli* XL1 Blue cells, and colonies expressing the full-length Myomedin-loop variants were verified by plasmid sequencing.

**Figure 2:**
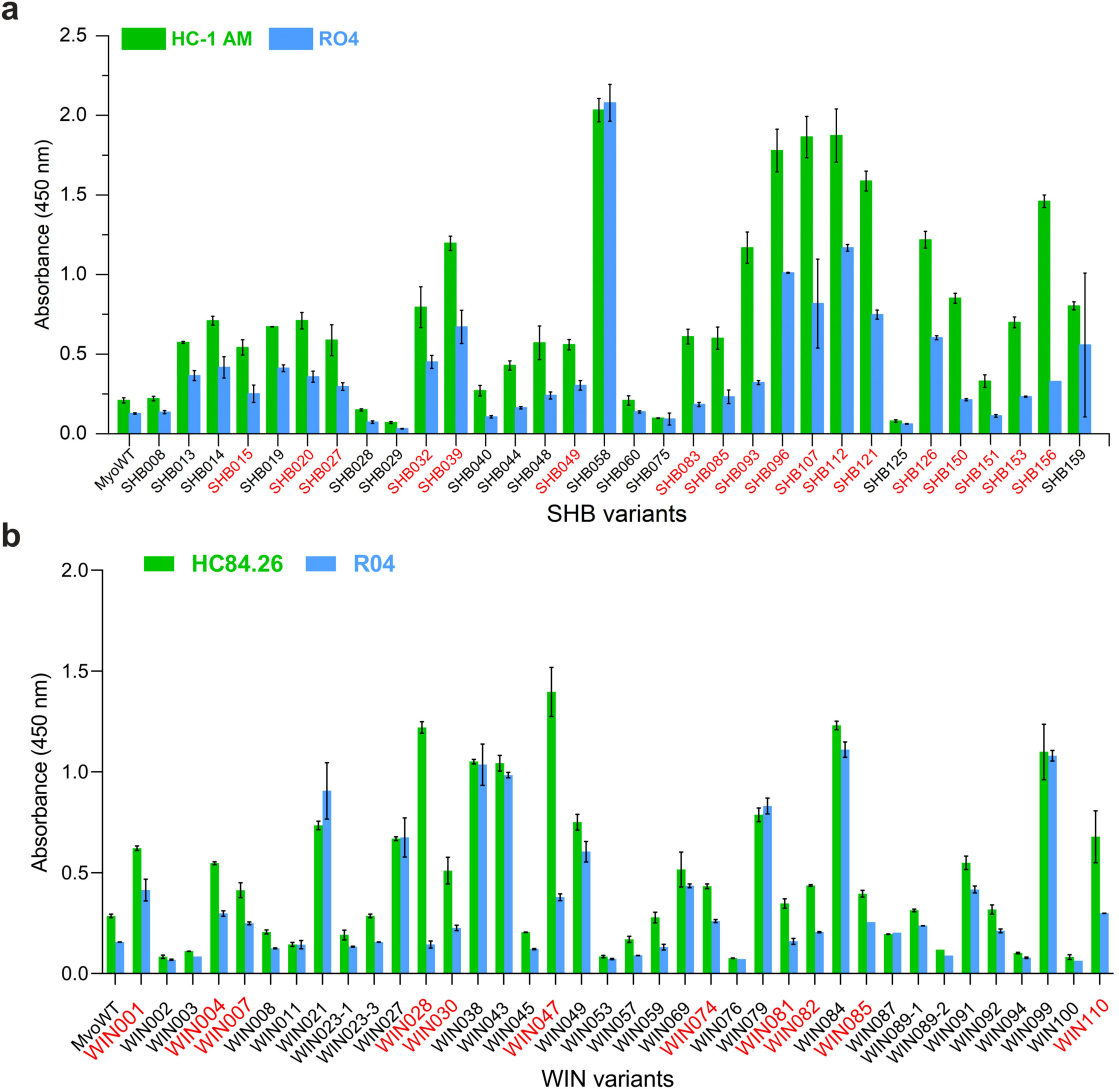
ELISA screening of bacterial lysates for binding to **(a)** HC-1AM (SHB variants) and **(b)** HC84 (WIN variants) antibodies (green color). As a negative control, isotype R04 antibody (blue color) was used. Specific binding was detected using anti-V5-HRP conjugated antibody. Variants selected for further analysis are indicated in red color.

**Figure 3:**
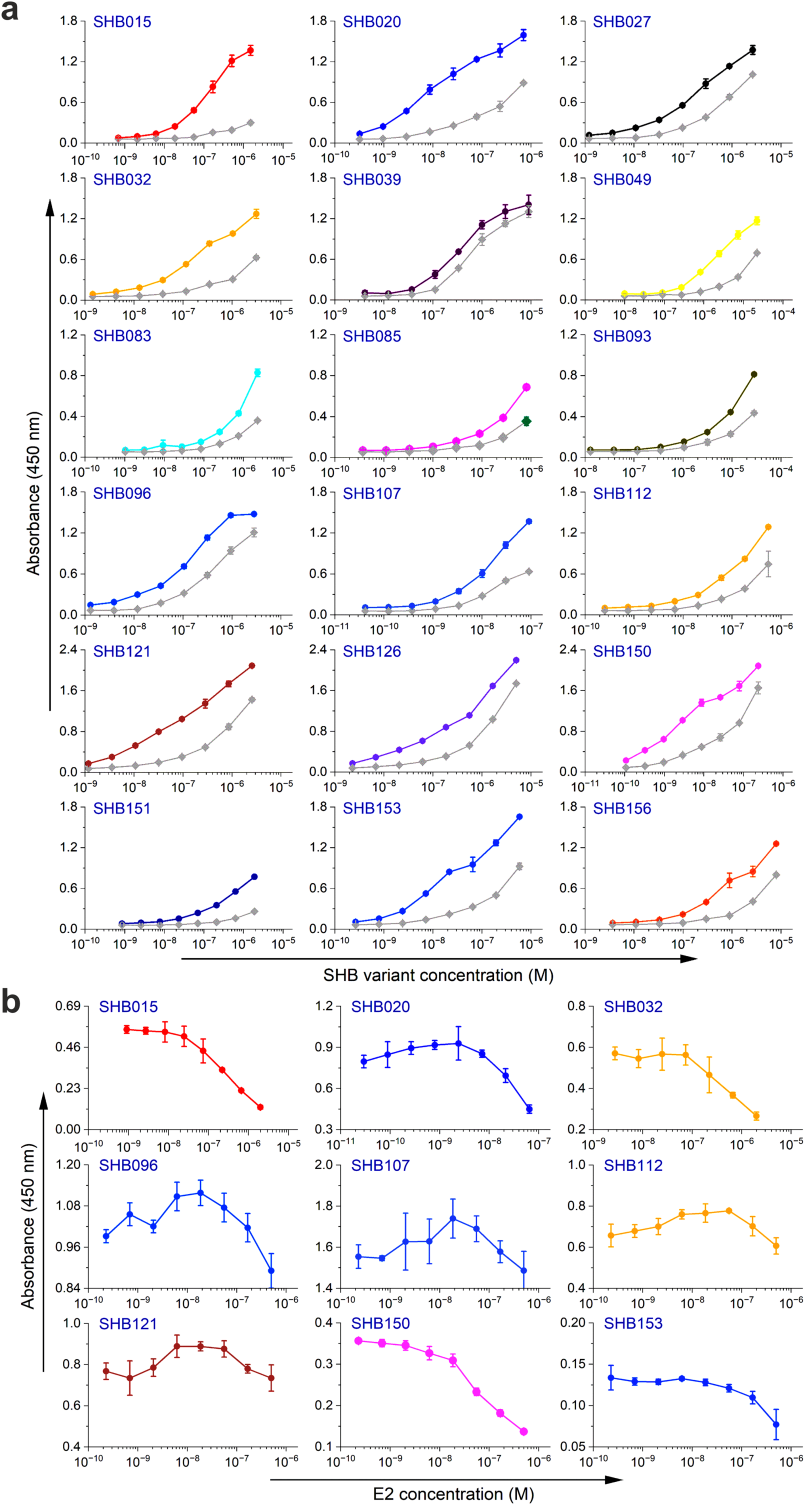
Binding and competition ELISA of SHB variants to the HC-1AM antibody: **(a)** Purified SHB variants were incubated with either the HC-1AM bNAb or the isotype control (human IgG1 λ or anti-HCMV R04 antibody) coated onto an ELISA plate. Specific binding was detected using an anti-V5 HRP-conjugated antibody. The colored lines show the specific binding of SHB variants to HC-1AM and the gray colored lines show the binding of SHB variants to the isotype control. **(b)** In the competition ELISA, serial dilutions of E2 protein were incubated with a constant concentration of SHB variants. The binding of the variants to the specific target antibody was detected using an anti-V5 tag antibody.

**Figure 4:**
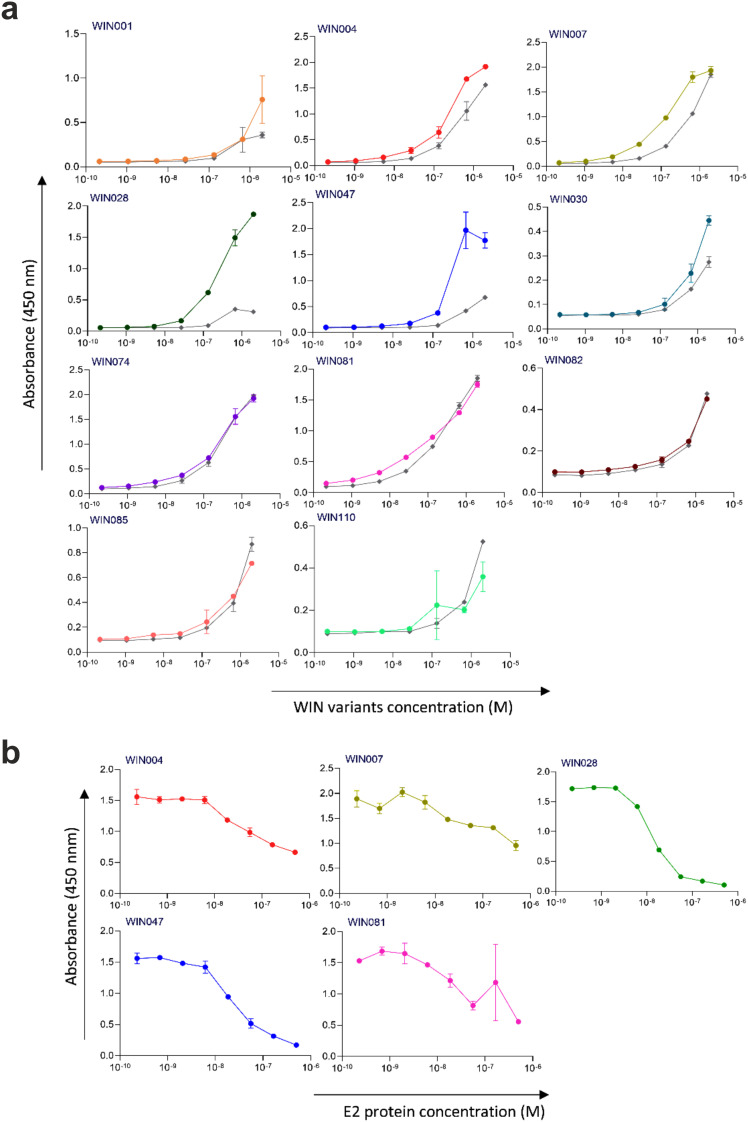
Binding and competition ELISA of WIN variants to the HC84 antibody: **(a)** Purified WIN variants were incubated with either the HC84 bNAb or the isotype control (human IgG1 λ or anti-HCMV R04 antibody) coated onto an ELISA plate. Specific binding was detected using an anti-V5 HRP-conjugated antibody. The colored lines show the specific binding of WIN variants to HC84 and the gray colored lines show the binding of WIN variants to the isotype control. **(b)** In the competition ELISA, serial dilutions of E2 protein were incubated with a constant concentration of WIN variants. The binding of the variants to the specific target antibody was detected using an anti-V5 tag antibody.

Next, the selected variants were expressed as 16 kDa Myomedin proteins, each containing a hexahistidine (His_6_)-tag at the N-terminus and a V5-tag at the C terminus (His-Myomedin-V5) in *E. coli* BL21 cells. Then, bacterial lysates of these selected variants were tested in an ELISA for binding to immobilized target antibodies, i.e., HC-1AM and HC84, with corresponding variants designated as SHB and WIN variants, respectively. During the cell lysate screening, Myomedin variants were selected based on their specific binding to the target antibody in comparison to the isotype control. Then, based on sequence analysis, only variants with mutations in the randomized positions in the loop regions were considered as structurally relevant Myomedins while those with the additional mutations were eliminated from the collection. A total of 29 variants (18 SHB and 11 WIN) were identified with considerable binding (≥ 0.2 a.u. of the absorbance) to the target-specific antibodies in comparison to the isotype control (**Figure 2**). These variants were subsequently produced and purified using a Ni-NTA agarose matrix. Next, the target-specific binding of Myomedin variants was assessed in the absence and presence of the HCV E2 protein as a competitor using ELISA. Serially diluted Myomedin binders (2 μg/ml) were used for ELISA binding curves generation, while a constant concentration (0.5 or 0.3 µM) of the Myomedins was mixed with serially diluted competitor E2 protein (0.5 or 0.3 µM) in competition ELISA. During the binding curve analysis, as shown in **Supplementary Figure S1**, SHB121 exhibited higher specific binding to HC-1AM in comparison to SHB027, SHB126, and SHB156 at lower concentrations (in the range 10–^9^ to 10–^7^ M). As shown in **Figures 3a** and **4a**, 9 SHB candidates (015, 020, 032, 096, 107, 112, 121, 150, and 153) out of 18 and 5 WIN (004, 007, 028, 047, and 081) variants out of 11 exhibiting specific binding to the target antibodies in comparison to the isotype control (human IgG1λ or anti-HMCV R04 antibody) were considered for competition ELISA.

**Figure 5:**
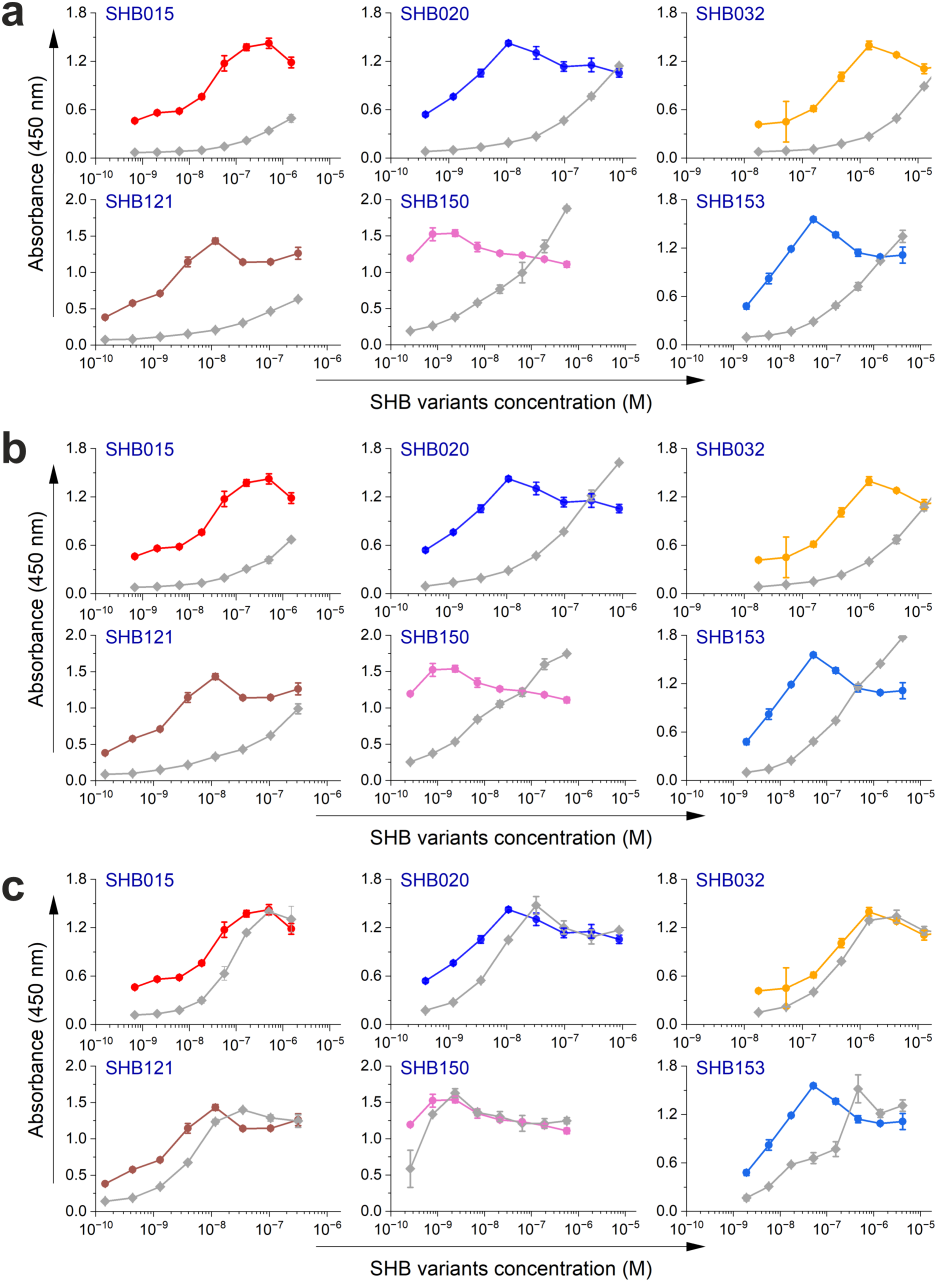
Cross-binding of selected SHB variants with **(a)** CBH-2, **(b)** HC33, and **(c)** HC84 HCV antibodies. Purified SHB variants were serially diluted and added into wells coated with HC-1AM antibody and antibodies selected for cross-binding. Specific binding was detected using an anti-V5 HRP-conjugated antibody. The color lines show specific binding of SHB variants to HC-1AM and gray color lines show the binding of SHB variants to antibodies considered for cross-binding.

**Figure 6:**
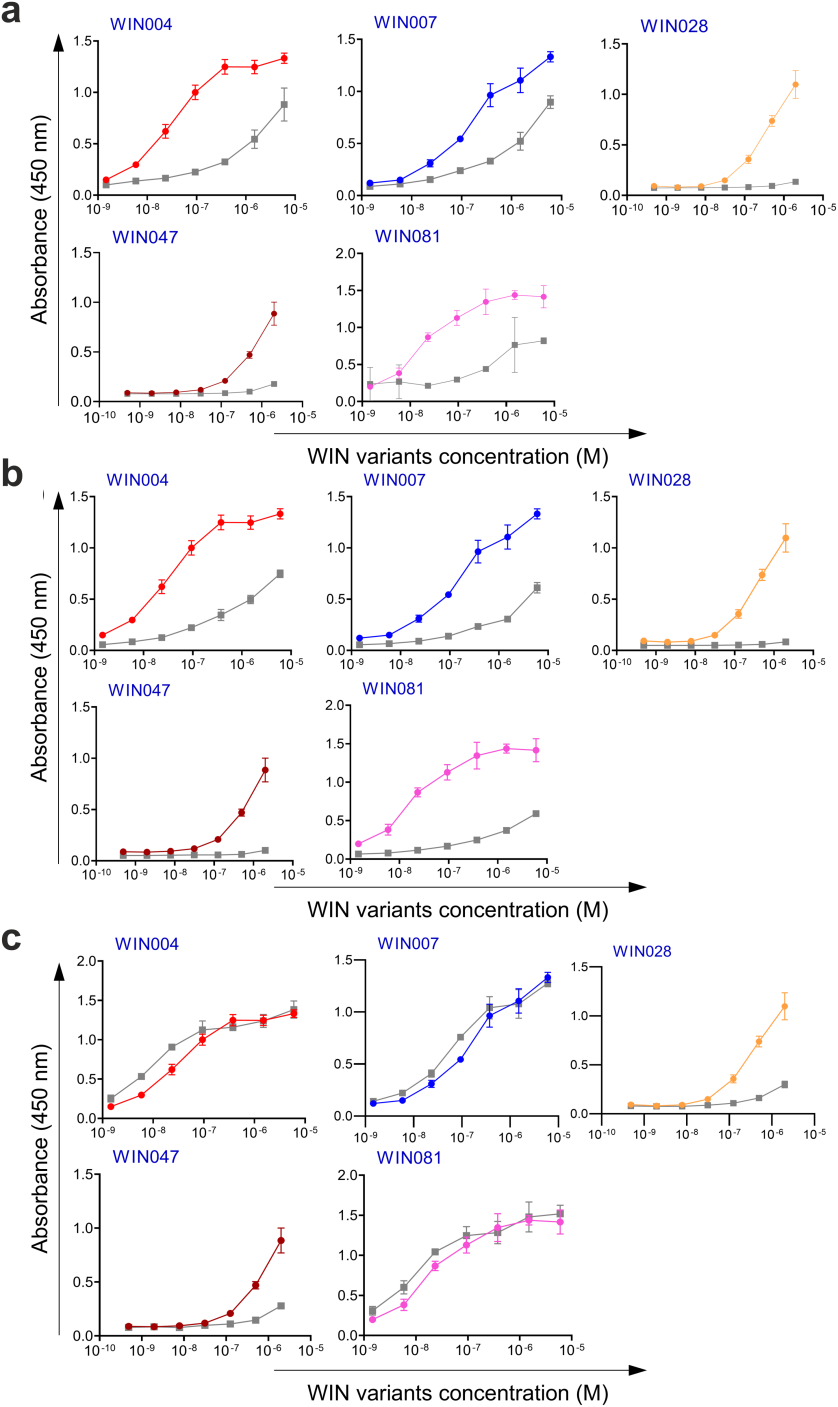
Cross-binding of selected WIN variants with **(a)** CBH-2, **(b)** HC33, and **(c)** HC-1AM HCV antibodies. Purified WIN variants were serially diluted and added into wells coated with HC84 antibody and antibodies selected for cross-binding. Specific binding was detected using an anti-V5 HRP-conjugated antibody. The color lines show the specific binding of WIN variants to HC84 and gray color lines show the binding of WIN variants to antibodies considered for cross-binding.

Additionally, as shown in **Figures 3b** and **4b**, the selected variants also exhibited considerable competition with HCV E2 protein at higher concentrations, reducing its binding to the target bNAbs, thereby confirming these variants as effective and selective mimics of HC-1AM and HC84 bNAbs. To further verify the specificity of the selected binders, we generated ELISA binding curves for the SHB variants, selected for bNAb HC-1AM, against HCV bNAbs targeting different domains of E2 (**Figure 5**). Results showed that SHB variants do not substantially bind to CBH-2 antibody (HCV E2 Domain B) and HC33.1.53 (HC-33) antibody (Domain E), although they exhibit considerable binding to HC84 antibody (Domain D). The overlap between Domain D and Domain B on the viral E2 protein may explain the binding of SHB variants to HC84. Similarly, cross-binding ELISA curves were also generated for WIN variants specific to bNAb HC84. As shown in **Figure 6**, WIN variants do not substantially bind to CBH-2 or HC-33 antibodies. The variants WIN004, WIN007 and WIN081 also bind to the HC-1AM antibody. Interestingly, the WIN028 and WIN047 variants exhibit specific binding to the HC84 antibody, suggesting that they mimic a Domain D epitope outside the region overlapping with Domain B. Therefore, based on the comparative binding and competition data analysis, six SHB proteins (SHB015, SHB020, SHB032, SHB121, SHB150, and SHB153) and five WIN variants (WIN004, WIN007, WIN028, WIN047, and WIN081) were selected for mouse immunization experiments.

### Myomedin mimotopes induce HCV E2 protein- and HCV pseudovirus-specific serum antibodies

**Figure 7:**
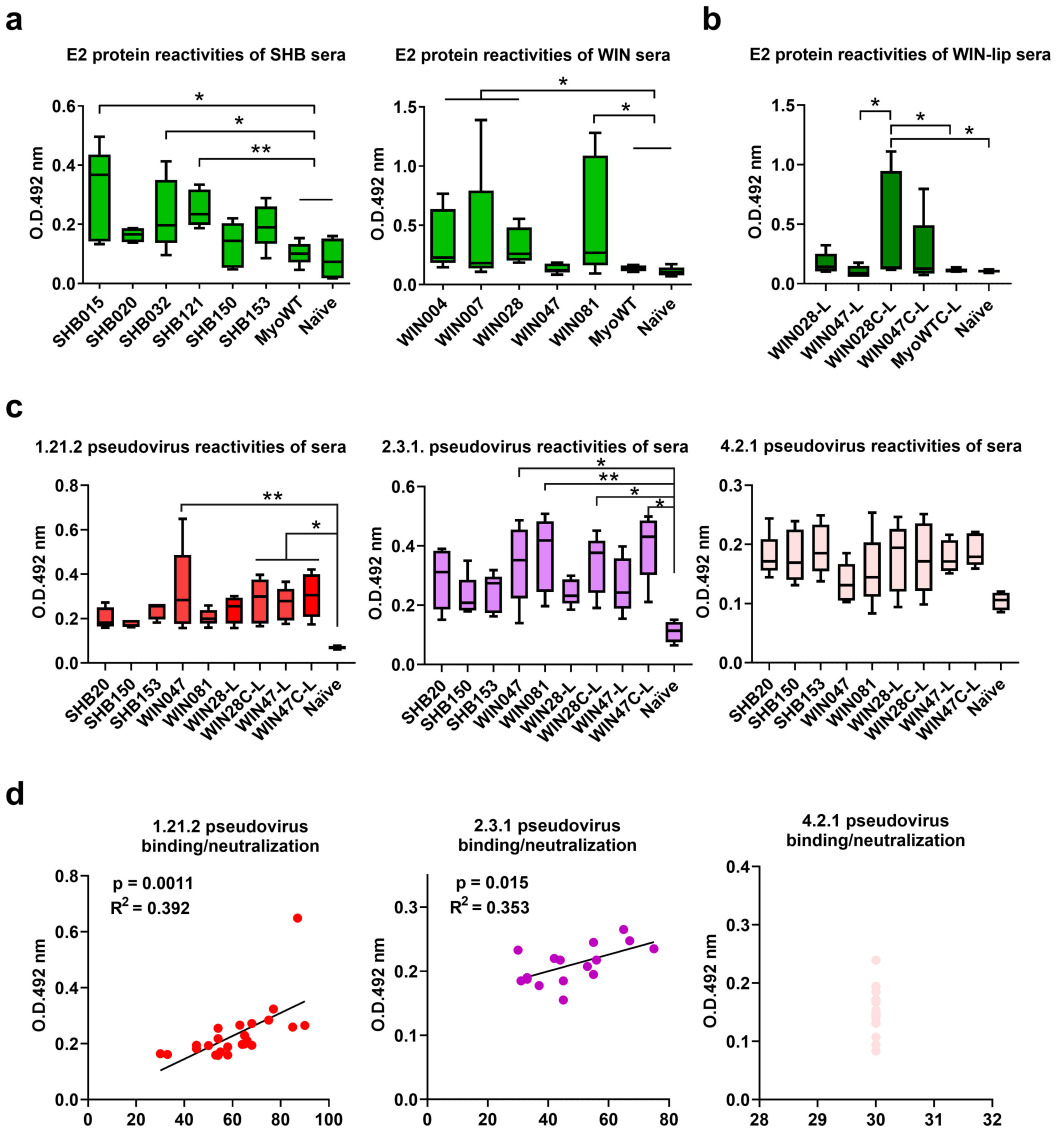
Analysis of binding antibodies from mouse hyper-immune sera. Experimental mice were immunized intradermally with **(a)** SHB, WIN proteins and **(b)** with proteoliposomal formulation of WIN028 and WIN047 with His-tag on N- (WIN028-L, WIN047-L) or C-terminus (WIN028C-L, WIN047C-L). Sera from **(a)** soluble SHB-immunized, **(b)** soluble WIN-immunized, and **(c)** WIN-proteoliposome immunized mice were collected two weeks after last immunization and the serum IgG reactivities with **(a, b)** HCV E2 protein and **(c)** UK Nottingham Panel (23) HCV pseudotyped viruses 1.21.2 (Genotype 1), 2.3.1 (Genotype 2), and 4.2.1 (Genotype 4) immobilized on 96-well plates were determined by ELISA. Statistical significance was calculated using the ANOVA Kruskal-Wallis model with Dunn’s post-test (* p < 0.05, ** p < 0.01). **(d)** Linear regression analysis of WIN-proteoliposomes immunized murine serum IgGs binding and the neutralization breadth toward 1.21.2., 2.3.1, and 4.2.1. pseudoviruses.

After *in vitro* selection and characterization, we assessed the immunogenicity of two groups of Myomedin mimotopes specific to HC-1AM (SHB015, SHB020, SHB032, SHB121, SHB150, and SHB153) and HC84 (WIN004, WIN007, WIN028, WIN047, and WIN081). The proteins were mixed in Freund’s adjuvant and administered intradermally. Mice hyperimmune sera were evaluated using an E2 antigen-specific ELISA after terminal bleeding. At a 1:50 dilution, immune sera showed that all Myomedin mimotopes induced a weak to moderate HCV E2-specific serum antibody response (**Figure 7a**). Mice administered with SHB015, SHB032, and SHB121 (specific to bNAb HC-1AM) showed significant immune activation when compared to naïve mice and Myomedin wild-type (MyoWT) immunized mice. Among the bNAb HC84 binders, variants WIN004, WIN007, WIN028, and WIN081 elicited significantly higher serum antibody titers targeting the HCV E2 protein (**Figure 7a**).

### Neutralization assay of anti-E2 antibodies against HCV pseudo particles

**Figure 8:**
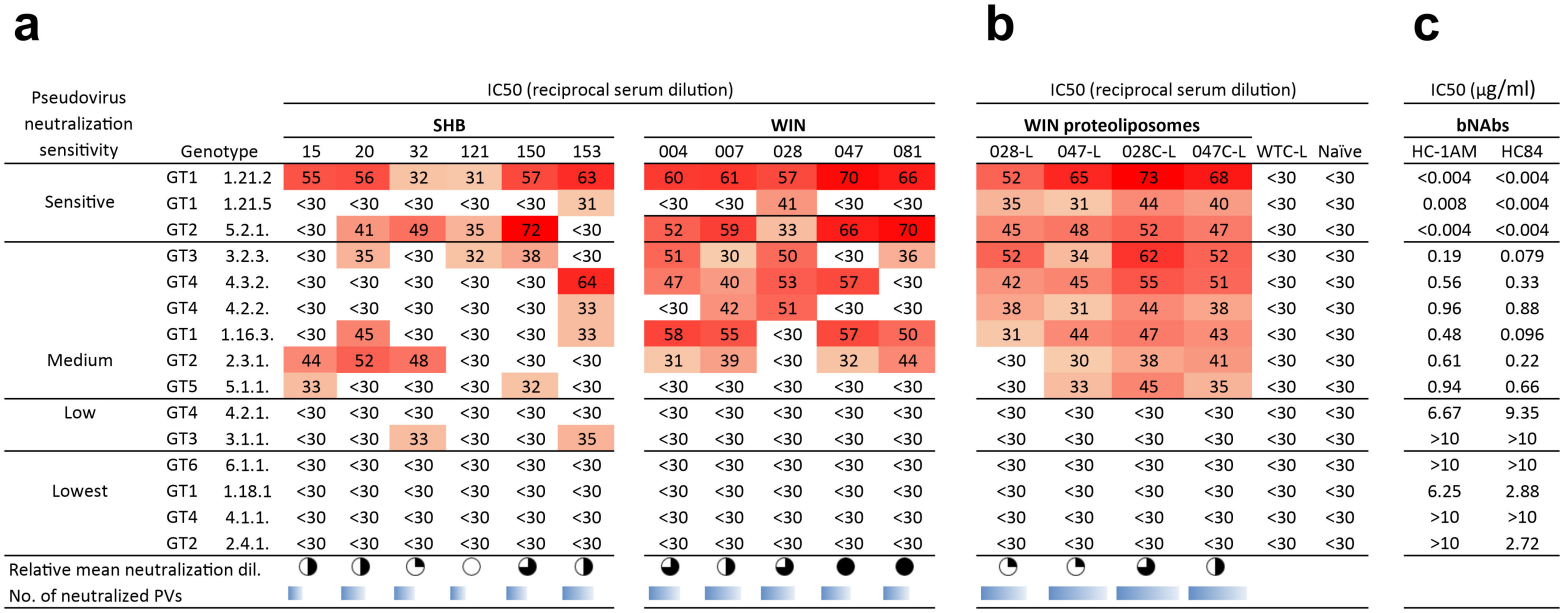
Neutralization capacity of hyper-immune sera of mice immunized with SHB and WIN. Sera from mice immunized with **(a)** soluble WIN and SHB proteins and **(b)** select WIN proteoliposomes were tested against a panel of 15 HCV-pseudotyped HIV viruses. 50% virus neutralization was reached by reciprocal serum dilution as shown by the color-coded values. The relative mean neutralization dilution reflects the average titers of the pseudoviruses used. The sum of the neutralized pseudoviruses for each Myomedin-induced sera variant is represented as the number of neutralized PVs and is calculated at reciprocal titers higher than 30. **(c)** Shows the comparison between the neutralization of bNAbs HC-1AM and HC84. The pseudoviruses were selected to include representatives from the most to the least neutralization sensitive HCV strains from the Nottingham Panel (23).

Next, we assessed the neutralization activity against a panel of diverse HCV pseudoviruses (PVs) from genotypes 1-6, exhibiting varying sensitivities to neutralizing antibodies, ranging from sensitive to extended resistance (23). As expected, the highest reactivity was observed against the most sensitive genotype 1 and 2 HCV pseudoviral strains. Based on the number of neutralized pseudovirus strains, the most efficient hyper-immune sera were induced by SHB020 (neutralized 5 PVs), SHB153 (neutralized 6 PVs), and WIN004 (neutralized 6 PVs). Additional sera, including WIN007, WIN028, WIN047, and WIN081, neutralized 5–7 PVs (**Figure 8a**). In the case of pseudoviral strains with medium neutralization sensitivity, binders targeting the HC84 epitope showed broader reactivity compared to binders mimicking the HC-1AM epitope.

### Optimization of WIN028 and WIN047 proteins for binding to liposomes and neutralization activities of hyperimmune sera

To enhance the intensity of the immune response, we generated WIN proteoliposomes as corpuscular vaccines. Proteoliposomes can provide higher-valency interactions between surface immunogens and B cell receptors during B cell activation. Thus, we used Ni-NTA lipids-carrying liposomes (26) to ensure an oriented presentation of His-tagged WIN binders. In this context the best performing binders, WIN047 and WIN028, were modified by reengineering the His-tag originally on the WIN N-terminus to WIN C-terminus (designated WIN028C and WIN047C) and assessed the effect of the oriented presentation of WIN binders on immunogenicity (24). The resulting WIN proteoliposomes (WIN028-L, WIN047-L, WIN028C-L, and WIN047C-L) were administered intradermally in three doses to experimental mice with MPLA adjuvant, replacing the Freund’s adjuvant used in non-liposomal experiments.

Hyperimmune sera from WIN proteoliposome immunized mice reacted in ELISA with recombinant HCV E2 protein (**Figure 7b**). Mice administered with the optimized WIN028C-L and WIN047C-L showed significant immune activation when compared to naïve mice and Myomedin wild-type proteoliposome immunized mice. The results indicate that linking WIN binders to proteoliposomes through the C-terminus improves the availability of these epitopes to elicit antibodies in comparison to N-terminally linked WIN. Three pseudoviruses representing high (1.21.2), medium (2.3.1), and low (4.2.1) neutralization sensitivity HCV variants (23) (**Figure 7c**) were selected for further analysis. We observed a broader neutralization of the tested HCV pseudovirus panel after WIN liposomal immunization (**Figure 8b**). Hyperimmune sera from N-terminally His-tagged WIN028-L and WIN047-L proteoliposomes neutralized 7 PVs and 9 PVs respectively, while the C-terminally His-tagged WIN028C-L and WIN047C-L proteoliposomes neutralized 9 PVs (**Figure 8b**). Furthermore, we analyzed the correlation between binding and neutralization serum antibodies in all proteoliposome-immunized mice (**Figure 7d**). Of note, these results show a good correlation between the binding and neutralization breadth (R^2^ = 0.392, *p* = 0.0011 and R^2^ = 0.353, *p* = 0.015; **Figure 7d**) for high and medium neutralization sensitive pseudoviruses 1.21.2 and 2.3.1, respectively. As expected, pseudovirus 4.2.1 with low neutralization sensitivity showed no correlation (**Figure 7d**). Hypothetically, a decrease in the exposure of neutralization epitopes may explain the reduced immunogenicity observed (27). Overall, the binders demonstrated similar reciprocal serum dilution, resulting in 50% pseudovirus neutralization of sensitive and medium sensitivity strains. Although we did not characterize the absolute concentrations of serum binding and neutralizing antibodies, we performed an approximate comparison using the same bNAbs, HC-1AM and HC84 used for binder selection (**Figure 8c**). Our murine sera exhibited minimal IC_50_ dependence on serum dilution for both sensitive and medium-sensitivity HCV pseudoviruses. For bNAbs, the differences in concentrations required to achieve IC_50_ for highly sensitive and medium sensitivity pseudoviruses is 200-fold higher while for highly sensitive and the lowest sensitive pseudoviruses is higher than 2,000-fold. This suggests that although the concentration of the antibodies elicited here was low, their activity and function against the sensitive and medium sensitivity pseudovirus panel was conserved after serial dilutions of the murine sera.

## Discussion

Pan-genotypic DAAs do not provide protection against HCV reinfections, are expensive, and have limited access especially in developing countries. Therefore, target product profiles for HCV vaccines must address these challenges. Furthermore, immunological correlates of protection from clinical-stage HCV vaccine trials suggest that novel strategies are required to improve vaccine efficacy (28). However, several obstacles remain in the quest to develop an efficacious HCV vaccine. Extensive glycosylation, exceptional conformational flexibility of conserved viral epitopes, and lipid shielding of the envelope glycoproteins are HCV immune evasion mechanisms that are challenging to overcome in vaccine design (29). In this study, we generated novel non-cognate Myomedin mimotopes by applying the combinatorial Myomedin-loop concept to the paratopes of HCV bNAbs, which successfully elicited anti-HCV E2 antibodies in a mouse model. This proof-of-principle approach to designing immunogens provides a vaccine scaffold that is highly stable and versatile. The Myomedin-loop library offers enhanced three-dimensional topology and conformational flexibility as non-cognate ligands to efficiently mimic HCV E2 viral epitopes. We previously showed that this approach accurately mimics the glycan-rich, highly dynamic V3 loop regions of HIV-1 gp120, despite not sharing sequence or glycan homology (20). Additionally, these Myomedins are derived from human Myomesin-1 domain 10 and are non-immunoglobulin proteins. Therefore, administering repeated injections of Myomedins poses a limited risk of unwanted immune responses in prime-boost vaccine strategies. Here, we use well-delineated bNAbs as templates to design mimotopes eliciting humoral immune responses to overlapping conserved conformational epitopes on the E2 neutralizing face and the CD81 binding loop (30, 31). Also, using a tiered genetically diverse HCV pseudo-virus panel, we demonstrate how this approach yields binders with the potential to neutralize clinically relevant strains (28, 32–34).

Spontaneous hepatic immune-mediated clearance of the primary HCV infection is driven by the early development of broadly neutralizing antibodies, and virus-specific CD4^+^/CD8^+^ T cell responses (29, 35). Interestingly, the durability of the primary immune response is insufficient to prevent reinfection. Evidence from human infections and nonhuman primate models suggests that antibody-induced clearance is vital for preventing the chronicity of HCV infections although sterilizing immunity is rare (30, 33, 36). Therefore, an ideal vaccine candidate for HCV should elicit sustained T cell responses and bNAbs targeting the conserved epitopes in the AR3 and discontinuous epitopes in antigenic sites 412 and 434 (15, 28, 37). The neutralizing domains targeted by the Myomedin binders in this study represent epitopes with low susceptibility to accumulate escape mutations and regions capable of eliciting protective adaptive immune responses (8, 38). Although our vaccine experiment did not directly measure T cell responses, analyses here confirm binding of the selected Myomedin variants to bNAbs HC-1AM and HC84 which are specific to the AR3 of the HCV E2 neutralizing face. Thus, a crucial component of an efficacious vaccine to prevent viral persistence can be inferred from these findings. Myomedin variants selected in this study are non-cognate ligands of HC-1AM and HC84 and were tested as non-continuous conformational epitopes for activating B cells and for the elicitation of E2-neutralizing face antibodies. Therefore, the detection of Myomedin-specific T cell responses in our experimental setup would only assess the interactions of the murine immune system with the Myomedin scaffold, and not the immune responses to the epitopes recognized by the elicited E2-NAbs nor the T cell protection against HCV infection. Our strategy utilizes non-cognate bNAbs ligands and focuses on the immune response to the neutralizing E2 front layer regions and not to the immunodominant non-neutralizing E2 back layer. In contrast, native (cognate) E1E2 immunogens elicit weak or non-neutralizing antibodies in human clinical trials (39). This has been attributed to structural flexibility of the E1E2 heterodimer which impedes access to conserved epitopes and easily neutralized conformational states (40). Moreover, antibody functionality is dependent on counteracting glycan epitope masking and conformational changes to the E2 domains, which are essential for binding and neutralization of AR3-directed bNAbs (15). In previous work against a densely glycosylated virus, Myomedin scaffold variants elicited bNAbs targeting conserved HIV-1 epitopes across several group M clades (20, 21, 41). Therefore, a limited conclusion based on the binding and neutralization data in this study can be made that by using Myomedin vaccine scaffolds, the glycan viral immune evasion mechanism utilized by HCV can be diminished.

Vaccine formulations and adjuvants are vital for inducing predefined cellular and humoral immune responses. Therefore, optimizing the binders might explain the moderate neutralization responses observed in the mice sera. Corpuscular modifications using a proteoliposomal formulation enhanced the breadth and potency of the observed murine immune response. Notably, the WIN binders exhibited improved immunogenicity due to the intrinsic properties of the proteoliposome formulation, the MPLA adjuvant, and the enhanced multivalency of the surface-presented WIN variants. The WIN binders targeting HC84 epitopes were selected for formulation and adjuvant modification based on their broader neutralization of HCV pseudoviruses demonstrating the versatility of these small proteins. However, the antibody and neutralization titers of the soluble proteins can still be improved to overcome the suboptimal intensity of immune responses observed. Other multivalent formulations of binders using particulate vaccines such as VLPs or multimerizing ferritin-binder fusion proteins could improve the intensity of the induced immune response. Further improvement could also be achieved by a combination of binders with antigens targeting CD8^+^ T cells such as nonstructural proteins that are dominant targets of CD8^+^ T cells (42). Also, the use of an appropriate nonhuman primate or humanized mouse model could better correlate the immune responses of HCV vaccine candidates (12). Results from clinical stage vaccine trials show improved immunogenicity through oil-in-water adjuvants and immunofocusing of the antigens (43).

Our immunogen design relies on protein mimicry of conformational epitopes in the E2 AR3. This is crucial for the design of protein-based HCV vaccine candidates. By mimicking conserved viral epitopes, potent bNAbs responses can be elicited in experimental models. However, the study did not assess the durability of the antibodies elicited as a viral challenge experiment was not conducted. Future studies incorporating T-cell epitopes and modified adjuvants within the vaccine design and a viral challenge could improve our understanding of the observed immune responses. In summary, we developed an HCV non-cognate Myomedin ligand-based vaccine that elicits potent broadly neutralizing antibodies targeting the viral E2 glycoprotein in mice.

## Data Availability

The raw data supporting the conclusions of this article will be made available by the authors, without undue reservation.
