# Supplementary material for: Non-cognate ligands of hepatitis C virus envelope broadly neutralizing antibodies induce virus-neutralizing sera in mice
Source: Front Immunol. 2025 Jul 22;16:1624299. doi: 10.3389/fimmu.2025.1624299 (PMC12321816; doi:10.3389/fimmu.2025.1624299)
Supplement: Supplementary file 1 [file DataSheet1.pdf]

Non-cognate ligands of hepatitis C virus envelope broadly neutralizing antibodies induce virus-neutralizing sera in mice

Stephen Ian Walimbwa[1,2†], Shiv Bharadwaj[3†], Petr Kosztyu[1], Lucie Vankova[3], Milan Kuchar[3], Eliska Kopecna[1], Roman Effenberg[4], Lukas Drasar[4], Leona Raskova Kafkova[1], Petr Maly[3*], Milan Raska[1*]

[1]Department of Immunology, Faculty of Medicine and Dentistry, Palacky University Olomouc and University Hospital, Olomouc, Czechia, [2]Infectious Diseases Institute, Makerere University College of Health Sciences, Kampala, Uganda, [3]Laboratory of Ligand Engineering, Institute of Biotechnology of the Czech Academy of Sciences, BIOCEV Research Center, Vestec, Czechia, [4]Department of Chemistry of Natural Compounds, University of Chemistry and Technology, Prague, Czechia

[†] These authors contributed equally

*Correspondence:

Milan Raska; Department of Immunology, Faculty of Medicine and Dentistry, Palacky University Olomouc, Hnevotinska 3, 779 00 Olomouc, Czech Republic, milan.raska@upol.cz

Petr Maly; Laboratory of Ligand Engineering, BIOCEV Research Center, Institute of Biotechnology of the Czech Academy of Sciences, Prumyslova 595, Vestec 252 50, Czech Republic, petr.maly@ibt.cas.cz

Figure S1: The binding of selected SHB variants to the immobilized HC-1AM monoclonal antibody and R04 isotype IgG control monitored by anti-V5-tag antibody conjugate in ELISA

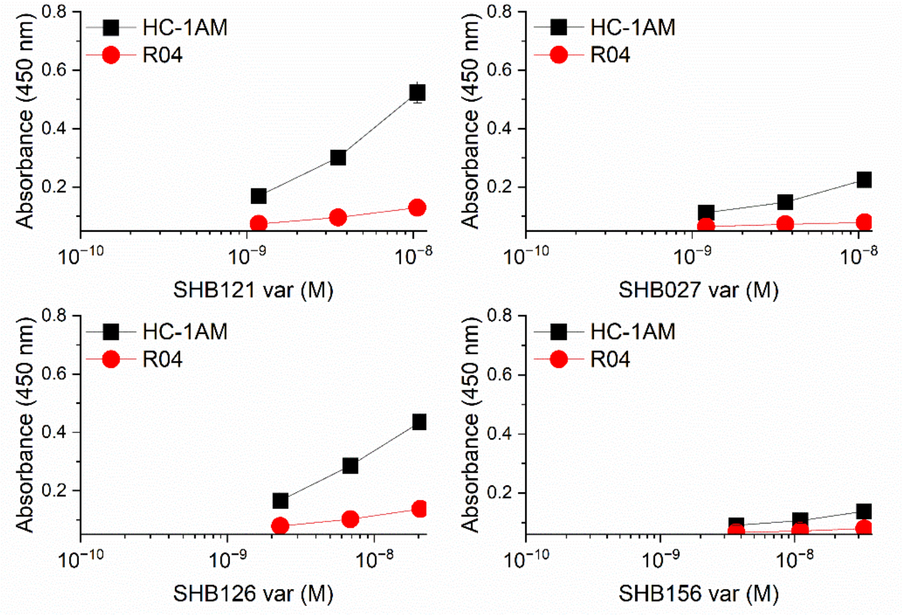

SHB121 exhibits higher specific binding to HC-1AM in comparison to SHB027, SHB126, and SHB156 at lower concentrations (in the range 10^{-9} to 10^{-7} M).
